# Adipocyte metabolism of the chemotherapy daunorubicin

**DOI:** 10.18632/oncoscience.428

**Published:** 2018-06-25

**Authors:** Steven D. Mittelman, Etan Orgel

**Affiliations:** Children's Discovery Institute, UCLA Mattel Children's Hospital, David Geffen School of Medicine, Los Angeles, CA 90095, USA

**Keywords:** obesity, anthracycline, pharmacokinetics, adi-posity, aldo-ketoreductase

It is now evident that obesity increases the risk of cancer mortality [1]. Much attention to this association has focused on how excess adipose tissue could increase cancer incidence; evidence points toward a number of factors, including increased levels of growth factors such as IGF-1, greater inflammation, and adipocyte production of estrogens [2]. However, in addition to its effects on cancer development, obesity is associated with worse survival from several cancers, including leukemia [3], breast, colon, and prostate [4]. The mechanisms whereby obesity impacts cancer outcome remain unclear.

While the pathophysiology of the interaction has yet to be fully elucidated, there is abundant evidence that fat cells exert a “local” effect on tumor cells. Adipocytes are abundant in many common tumor microenvironments, such as the bone marrow, breast, and omentum. Some cancers even induce the development of adipocytes within their tumor microenvironment. These localized interactions within the microenvironment provide the opportunity for adipocytes to promote the survival of cancer cells through a variety of mechanisms, which generally fall into two categories: fuel supply and survival signaling. Adipocytes directly release a number of fuels which could be important for cancer cells, including free fatty acids (FFA), glycerol, glutamine and other amino acids, and lactate. Increased fuel availability could promote cancer cell growth and replication, increase tumor size and genetic heterogeneity, and maintain cellular oxidative balance in the face of cytotoxic chemotherapy. For leukemia specifically, adipocyte provision of asparagine and glutamine could directly counteract the metabolic targeting effect of L-asparaginase [5]. With respect to survival signals, adipocytes are known to secrete a number of cytokines and adipokines which may contribute to cancer cell survival. Leptin, IL-6, adiponectin, and resistin are a few of these factors with putative roles in cancer cell progression. Additionally, adipocytes near cancer cells often develop an altered phenotype, characterized by loss of lipid stores and lower expression of adipocyte markers. These “cancer-associated adipocytes” appear to further support cancer cells via excess release of free fatty acids, increased secretion of pro-inflammatory cytokines, and release of proteases which may support tumor invasion and metastasis [6].

In addition to these paracrine effects, obesity and adiposity have systemic effects on the body. Obesity induces a state of insulin resistance, resulting in increased levels of circulating fuels such as glucose, FFA, triglycerides, and branched chain amino acids. Obese individuals also have increased circulating levels of hormones such as insulin, free insulin-like growth factor-1 (IGF-1), and ghrelin, which could further promote cancer cell survival. Thus, the obese state is associated with alterations in local and systemic levels of metabolites, hormones, and cytokines, which together could promote a pro-tumor environment. These factors could enhance tumor initiation, invasion and metastasis, chemotherapy resistance, and regrowth of surviving cells leading to relapse.

Our group has been primarily interested in how obesity and adipocytes might impair the efficacy of chemotherapy for pediatric acute lymphoblastic leukemia, the most common childhood cancer. We hypothesized that the interaction of obesity and chemotherapy is multifactorial, but includes changes to chemotherapy delivery to the cell from obesity-altered pharmacokinetics/pharmacodynamics (PK/PD) in the host. The effects of obesity on chemotherapy delivery and metabolism are not well-studied, with a paucity of data to guide dosing practices for the increasingly common obese patient [7]. Historically, despite the scarcity of data, clinical practice frequently adjusted chemotherapy dosing for obese patients, such as dosing by ideal body weight; this practice continues for some lipophilic agents such as the common chemotherapy agent vincristine.

In our recent study, we describe a new mechanism whereby adipocytes may contribute to cancer relapse and poorer survival: adipocytes absorb anthracycline chemotherapies, effectively sequestering them from the local tumor microenvironment [8]. While it has long been known that adipose tissue accumulates liphophilic compounds, we observed that the anthracycline daunorubicin (DNR) accumulated in the cytoplasm, not lipid droplets of the adipocytes. Further, we made the novel discovery that adipocytes metabolize DNR into the largely inactive metabolite, daunorubicinol. This is the first description to our knowledge of adipocytes metabolizing a pharmaceutical drug, and the first instance of adipocyte-mediated changes in microenvironment PK/PD affecting drug delivery to the leukemia cell. This process appears to occur due to the very high expression in adipocytes of several aldo-keto reductase and carbonyl reductase enzymes known to metabolize anthracyclines.

Adipose tissue has been much maligned in recent decades due to the obesity epidemic and its public health implications. However, it is important to keep in mind the importance of this tissue. Adipose tissue undoubtedly evolved to protect organisms from “the thousand natural shocks that flesh is heir to.” Fat tissue physically cushions us from trauma, defends our body from hypothermia, and provides needed fuels in times of food scarcity. It might not be surprising therefore that adipose tissue protects and supports malignant as well as non-malignant cells. Greater understanding of the mechanisms of this support might enable a path toward reversing adipocyte protection of cancer cells to maximize chemotherapy delivery, cytotoxicity, and improve survival for patients.
